# A Rare Case of Susac's Syndrome Masquerading as Progressive-Relapsing Multiple Sclerosis

**DOI:** 10.7759/cureus.25366

**Published:** 2022-05-26

**Authors:** Nedal Darwish, Halima Bakillah, Emily Rey, Kyle Berliner, Dayakar Reddy

**Affiliations:** 1 Internal Medicine, Arnot Ogden Medical Center, Elmira, USA; 2 Radiology, Arnot Ogden Medical Center, Elmira, USA

**Keywords:** visual field defect, sensorinerual hearing loss, autoimmune vasculitis, multiple sclerosis and other demyelinating disorders, internal medicine and rheumatology, susac syndrome

## Abstract

Susac’s syndrome (SS) is a rare, autoimmune-mediated vasculitis involving central nervous system (CNS) microvasculature, which typically targets the brain, retina, and cochlea. The disease pathology in these regions produces the characteristic triad of encephalopathy, visual loss, and hearing loss. Unfortunately, less than 20% of cases present as the full triad, often making diagnosis challenging. Diagnosis is also confounded by the similarity in the clinical presentation of multiple sclerosis (MS), with an overlap also seen in laboratory studies and radiographic imaging. In this report, we present a case of SS in a young and previously healthy adult male that was initially mistaken as MS. We review the characteristics of SS and highlight the key differences between the two diseases that can be used by diagnosing physicians. Lastly, we describe the treatment strategies involved in SS compared to MS.

## Introduction

Susac’s syndrome (SS) is a rare, autoimmune-mediated vasculitis involving central nervous system (CNS) microvasculature [1]. This disease typically targets the brain, retina, and cochlea, giving the characteristic triad of encephalopathy, vision loss, and hearing loss. Less than 20% of cases present as the full triad, often making diagnosis challenging. The purpose of this case report is to discuss the difficulty in diagnosis of SS and highlight the key clinical differences between the presentation and diagnostic studies of SS and multiple sclerosis (MS) that can be used by diagnosing physicians. As the treatment of SS and MS can be starkly different, misdiagnosis and subsequent treatment failure can lead to irreversible neurological damage to the patient. As this disorder has an “orphan disease” status, adding our patient’s clinical course to the known SS literature is vital. This case describes a 21-year-old male who presents with neurological complaints that are originally misdiagnosed as MS before SS is correctly diagnosed. 

## Case presentation

A previously healthy 21-year-old male presented to the emergency department (ED) with complaints of slurred speech, blurry vision, left-sided weakness, and progressive memory loss. He first noticed these symptoms four weeks prior to presentation and described them as intermittent in nature and without any obvious triggers or associations. The duration of the symptoms was variable, lasting anywhere from several days to weeks with large periods in between when the patient was symptom-free. At times, the visual dysfunction and vertigo became so severe that they resulted in multiple falls. Vital signs were normal as was the physical exam aside from the neurological portion, which revealed an ataxic gait, recall memory of 2/3 objects, anomic aphasia, and dysdiadochokinesia with poor finger to nose testing that was worse on the left side. All findings were highly unusual for his age and unremarkable medical history.

**Figure 1:**
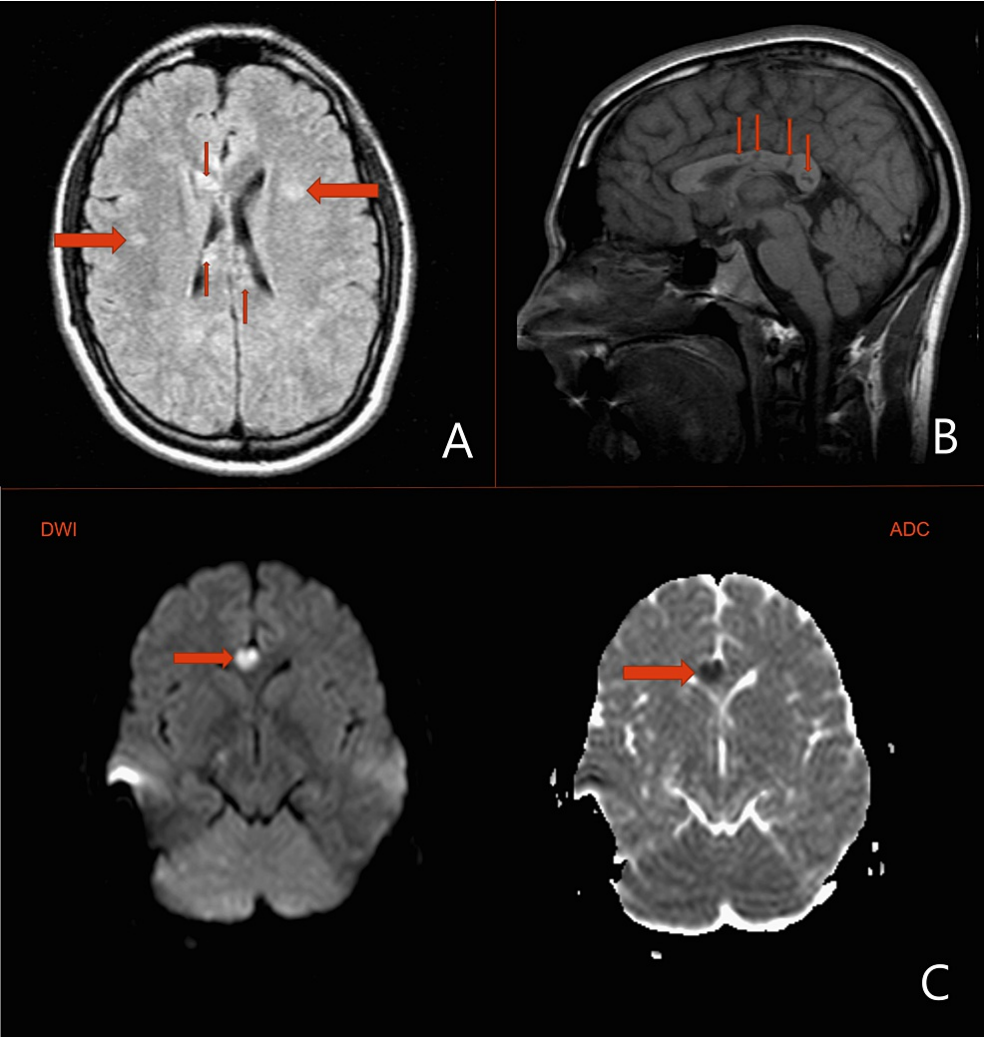
MRI imaging of Susac's syndrome in a 21-year-old male (A) T2-FLAIR imaging demonstrating scattered hyperintensities in both cerebral hemispheres and periventricular white matter. (B) T1 imaging demonstrating classic punched-out lesions located centrally in the corpus callosum. (C) DWI (left) and corresponding ADC (right) images demonstrating restricted diffusion and infarction of the corpus callosum. MRI: Magnetic resonance imaging; T2-FLAIR: T2-weighted-fluid-attenuated inversion recovery; DWI: Diffusion-weighted imaging; ADC: Apparent diffusion coefficient.

The initial workup consisted of a noncontrast CT scan of the head, complete blood count (CBC), comprehensive metabolic panel (CMP), thyroid-stimulating hormone (TSH), ammonia levels, toxicology screen, and urine analysis studies, which were remarkable. MRI of the head and neck revealed multifocal bilateral infarcts in both lobes of the cerebellum, cerebral hemispheres, and periventricular white matter (Figure 1, Panels A and B). Lesions were most intense at the corpus callosum suggesting a small area of encephalomalacia (Figure 1, Panel C). The radiologist interpreted these as findings that could be consistent with various conditions including a possible infectious process, a demyelination syndrome such as MS, or a cerebral vasculitis process of unclear etiology.

Lumbar puncture was performed, and the patient's cerebral spinal fluid (CSF) was found to have a markedly elevated nucleated cell count of 24 cells/uL (normal: 0-5 cells/uL) with 82% lymphocytes and an elevated protein level of 225 mg/dL (normal: 15-45 mg/dL). CSF gram stain, fungal, and viral panel, however, resulted as negative for any organisms, all but ruling out an infectious process as the underlying cause. No evidence of oligoclonal banding was observed, indicating that this may not be a demyelinating process. However, upon further analysis, myelin basic protein in the CSF was found to be elevated at 7.9 ug/L (normal: 0-4 ug/L) indicating there was indeed a breakdown of the myelin sheath, which is most often seen in MS. Further laboratory studies revealed a normal erythrocyte sedimentation rate and C-reactive protein, negative antinuclear antibodies, herpes simplex virus 1/2, Lyme, and Borrelia titers. The venereal disease research laboratory (VDRL) test was non-reactive, and the folate/B12 levels were normal. Only one set of MRI images was obtained, and the patient was diagnosed with MS based on the clinical symptomology, radiographic images, and CSF findings consistent with this disease. No other studies were considered to confirm the diagnoses as per the consulting neurologist.

Treatment consisted of a three-day course of pulsed IV methylprednisolone, which rapidly improved the patient's weakness and ataxic gait and completely resolved the blurry vision. The patient was discharged on a 14-day steroid taper with an outpatient follow-up appointment at the neurology clinic. Four weeks later, the patient returns with worsening blurry vision and new-onset hearing loss. A visual field exam revealed superior field deficits in nasal and temporal fields in the left eye. Fundoscopy of the left eye showed a hypopigmented retina surrounding the optic disc. The ophthalmology service was consulted and confirmed a branch retinal artery occlusion. Repeat laboratory studies did not reveal a coagulopathy of any sort, and additional testing for thrombotic disorders such as antiphospholipid syndrome was deemed unnecessary for the time being. Additionally, an audiometry screen revealed a low-frequency hearing loss in the left ear. This specific constellation of findings led to the diagnosis of demyelinating process stemming from a rare autoimmune-mediated CNS vasculitis known as Susac’s syndrome.

## Discussion

Susac's syndrome, also known as SICRET (small infarctions of cochlear, retinal, and encephalic tissue), is a rare neuroinflammatory disease with as few as 500 documented cases worldwide. Although the exact pathogenesis is unknown, it is thought to be due to autoimmune-mediated vasculitis pathology of the CNS microvasculature. Inflammatory attacks on small vessels in the CNS result in microvascular occlusion that can lead to irreversible microinfarction and neurovascular injury. The affected CNS microvasculature in this disease is specific to three locations: the brain, the cochlea, and the retina. This pathology manifests as the characteristic clinical triad of encephalopathy, sensorineural hearing loss, and vision loss due to branch retinal artery occlusion. This distinctive constellation of symptoms leads physicians to suspect SS as a differential diagnosis for CNS abnormalities. However, in addition to the rare incidence of this disease, patients rarely present with the complete triad of clinical findings, thus making it extremely difficult to diagnose.

Along with the imaging, clinical and laboratory findings can be used to competently differentiate between SS and MS as listed in Table 1. MRI of the brain is the diagnostic imaging modality of choice for SS. MRI findings include bilateral diffuse white matter lesions seen in multiple locations including but not limited to both cerebral hemispheres, periventricular white matter, brainstem, cerebellum, and corpus callosum [2]. The radiographic findings can be confounding as they are almost identical to those found in demyelinating disorders, especially MS. As listed in Table 1, there are key differentiating features that should increase the clinical suspicion for SS. Punched-out lesions located specifically in the central region of the corpus callosum is the most specific diagnostic finding in SS, a notable deviation from MS, in which the lesions are specific to the peripheral region of the corpus callosum [3].

**Table 1:** Distinguishing features of Susac's syndrome and multiple sclerosis CSF: Cerebral spinal fluid; MRI: Magnetic resonance imaging.

	Multiple Sclerosis	Susac’s Syndrome
Clinical presentation	Young adult female predominance	Young adult female predominance
	Visual loss: Optic neuritis	Visual loss: Branch retinal artery occlusion
	Headache (Uncommon)	Headache (Common)
	Hearing loss (Uncommon)	Hearing loss (Common)
	Encephalopathy (Uncommon)	Encephalopathy (Common)
Pathophysiology	Cell-mediated autoimmune attack on myelin with loss of oligodendrocytes	Cytotoxic T-cell-mediated autoimmune attack on microvascular endothelium
CSF analysis	Elevated protein (Uncommon)	Elevated protein (Common)
	Mild lymphocytic pleocytosis (Common)	Mild lymphocytic pleocytosis (Common)
	Oligoclonal bands (Common)	Oligoclonal bands (Uncommon)
MRI findings	Demyelination	Microinfarction
	Lesion characteristics: Larger ovoid, persist and scar, central vein sign, hyperintense with hypointense rim	Lesion characteristics: Smaller punctate, resolve without scarring, diffuse microvascular pattern, T2-hyperintense “snowball” appearance
	Locations of lesions: Corpus callosum-peripheral, periventricular cerebral white matter, cerebellum (Uncommon), spinal cord	Locations of lesions: Corpus callosum-central, periventricular cerebral white matter, cerebellum, brainstem, internal capsule

When these lesions in this anatomical region are found, suspicion for SS should be high, especially when concomitant clinical triad manifestations of the cochlea and retina are present as well. It has been noted that funduscopic findings of Gass plaques and arteriolar wall hyperfluorescence have been noted in SS [4]. Other clinical clues can include findings of headache, bilateral tinnitus, and hearing loss. Headache is seen in up to 80% of patients with SS and is relatively uncommon in MS [5]. Tinnitus and hearing loss will usually improve with time in SS but are permanent in MS. Also, tinnitus and hearing loss that occurs bilaterally is extremely rare in MS and should prompt a clinician to consider SS as a differential diagnosis.

Due to the rarity of this condition, the treatment course varies from case to case, typically based on the pattern of disease and clinical course. Acute symptoms are typically treated with high-dose IV steroids for a few weeks until symptoms resolve, followed by a several-month tapered course of oral steroids. SS can be self-limiting; however, as seen in the young male in this case, it often relapses with fluctuating symptoms similar to the relapsing-remitting pattern seen in MS. Therefore, continuous long-term treatment after the initial presentation is preferred to prevent a probable relapse of the disease. In SS with relapsing symptoms, long-term immunosuppressive therapy with cyclophosphamide or mycophenolate is deemed essential to prevent irreversible damage to the retina and cochlea. Irreversible damage in unsuppressed SS often manifests as the unfortunate permanent loss of hearing and vision as occurred in our patient, highlighting the importance of distinguishing between confounding differential diagnoses.

## Conclusions

Imaging, clinical, and laboratory findings can be used to competently differentiate between SS and MS. The triad of encephalopathy, visual loss, and hearing loss is a characteristic of SS. Punched-out lesions located in the central region of the corpus callosum in SS are a notable deviation from MS, in which the lesions are specific to the peripheral region of the corpus callosum. Treatment with long-term immunosuppressives in SS is essential to prevent irreversible damage to the retina and cochlea.
